# Vaccines against a Major Cause of Abortion in Cattle, *Neospora caninum* Infection

**DOI:** 10.3390/ani1030306

**Published:** 2011-09-08

**Authors:** Thierry Monney, Karim Debache, Andrew Hemphill

**Affiliations:** Institute of Parasitology, Vetsuisse Faculty, University of Berne, Länggass-Strasse 122, CH-3012 Berne, Switzerland; E-Mails: thierry.monney@vetsuisse.unibe.ch (T.M.); kardebache@yahoo.fr (K.D.)

**Keywords:** *Neospora caninum*, abortion, vaccination, host cell interaction, recombinant antigen, DNA-vaccine, live vaccine

## Abstract

**Simple Summary:**

We review the efforts to develop a vaccine against neosporosis, caused by the apicomplexan parasite *Neospora caninum*. Vertical transmission is the main mode of infection, and can lead to stillbirth, abortion, or birth of weak calves. We provide information on the biology of *Neospora caninum* and on the disease caused by this parasite, and summarize the current understanding on how the host deals with infection. We review studies on live- and subunit-vaccines, and demonstrate advantages and setbacks in the use of small laboratory animal models in investigations on a disease with high relevance in cattle.

**Abstract:**

Neosporosis, caused by the apicomplexan parasite *Neospora caninum*, represents one of the economically most important causes of abortion in cattle. During pregnancy, the parasite infects the placental tissue and the fetus, which can lead to stillbirth, abortion, or birth of weak calves. Alternatively, calves are born without clinical symptoms, but they can carry over the parasite to the next generation. In addition, *N. caninum* causes neuromuscular disease in dogs. The economic importance of neosporosis has prompted researchers to invest in the development of measures to prevent infection of cattle by vaccination. A good vaccine must stimulate protective cellular immune responses as well as antibody responses at mucosal sites and, systemically, must activate T-helper cells to produce relevant cytokines, and must elicit specific antibodies that aid in limiting parasite proliferation, e.g., by interference with host cell invasion, activation of complement, and/or opsonization of parasites to have them killed by macrophages. Different types of vaccines have been investigated, either in bovines or in the mouse model. These include live vaccines such as naturally less virulent isolates of *N. caninum*, attenuated strains generated by irradiation or chemical means, or genetically modified transgenic strains. Live vaccines were shown to be very effective; however, there are serious disadvantages in terms of safety, costs of production, and stability of the final product. Subunit vaccines have been intensively studied, as they would have clear advantages such as reduced costs in production, processing and storage, increased stability and shelf life. The parasite antigens involved in adhesion and invasion of host cells, such as surface constituents, microneme-, rhoptry- and dense granule-components represent interesting targets. Subunit vaccines have been applied as bacterially expressed recombinant antigens or as DNA vaccines. Besides monovalent vaccines also polyvalent combinations of different antigens have been used, providing increased protection. Vaccines have been combined with immunostimulating carriers and, more recently, chimeric vaccines, incorporating immuno-relevant domains of several antigens into a single protein, have been developed.

## 1. Introduction

*Neospora caninum* is an apicomplexan, which belongs to the family of Sarcocystidae. The parasite is distributed worldwide and infects a broad range of animals including cattle, sheep, goats, deer, horses and dogs. Due to its close resemblance to *Toxoplasma gondii*, which is of high medical as well as veterinary medical importance, it was recurrently misdiagnosed as such until Dubey and colleagues succeeded in isolating the parasite in the late 1980s [1]. *Neospora caninum* is the causative agent of neosporosis, a serious illness associated with abortion, stillbirth and maternal infertility in cattle and neurological disease in dogs. Dogs represent the definitive host of *N. caninum*, where sexual development takes place. In contrast to *T. gondii*, there is no evidence that *Neospora caninum* can be transmitted from animals to humans [2].

Infection with *N. caninum* elicits a humoral immune response. The prevalence of *N. caninum* varies considerably in different countries. In Europe, national seroprevalence levels fluctuate between 16–76% for dairy, and 41–61% for beef cattle [3]. Higher seroprevalence (97–100%) has been reported from dairy, beef and sheep farms in New Zealand [4]. A recent survey in China [5] showed that there is a connection between seroprevalence and animal husbandry practices, in that freely grazing cows exhibited a significantly higher seroprevalence compared to cattle feeding under controlled conditions. In Switzerland the average seroprevalence lies between 10–15% [6].

In terms of its economic impact for the livestock industry, bovine neosporosis is of considerable importance. Beside the loss caused by abortion itself, other factors to consider include reduced milk yield, premature culling and reduced post-weaning weight gain in beef calves. However, there are conflicting reports regarding the importance of milk yield and weight gain [7]. In California, an estimated 40,000 abortions are due to neosporosis, causing a loss of approximately 35 million US Dollars per year. In Australia and New Zealand, losses are thought to be more than 100 million Australian Dollars per year [4,8], and in the Netherlands estimates of 19 million Euros have been reported. A Swiss study calculated the annual losses due to *N. caninum* in the Swiss dairy cow population at 9.7 million Euros [9]. Therefore, the economic importance of neosporosis, especially in cattle, has lead to research on the development of strategies for prevention and treatment of an *N. caninum* infection. Vaccination and chemotherapy have been identified as economically promising options, provided that suitable targets and effective reagents would be identified [9,10]. In this review, we will focus on vaccines against neoporosis.

## 2. Biology of *N. caninum*

*N. caninum* is an obligatory intracellular parasite. Invasive stages must infect a host cell in order to survive, proliferate and proceed in the life cycle. This life cycle includes three distinct infective stages, which are named tachyzoites, bradyzoites and sporozoites. The acute infection is characterized by tachyzoites, which represent the rapidly proliferating forms that lyse their host cells within a few days following infection to release considerable numbers of progeny [11]. In response to immune pressure, the parasites differentiate into bradyzoites, which reside within an intracellular tissue cyst, mostly in the central nervous system and muscle tissues, and proliferate only slowly, thus marking the chronic stage of infection. Bradyzoites can persist within chronically infected animals for many years without any clinical symptoms. Ingestion of bradyzoite-infected meat by canids such as dogs, wolves and dingoes can result in sexual development within the intestinal tissue of the definitive host [12], and possibly shedding of oocysts that undergo meiosis, and subsequently form eight haploid sporozoites in the environment. Sporulated oocysts are orally infective for both definitive and intermediate hosts.

Tachyzoites represent the disease-causing stage. They can infect and proliferate within many different cell types and tissues, and the capacity to infect the cells of the reticulo-endothelial system such as macrophages, lymphocytes and dendritic cells ensures rapid dissemination of parasites into different organs [13,14]. Tachyzoites can also enter placental tissue, disturb the fragile immunological balance during pregnancy, and invade fetal tissue [15]. This often leads to stillbirth, abortion or birth of weak calves. *In utero*-transmission of *N. caninum* is highly efficient. However, in an immune-competent host, tachyzoites are attacked and largely eliminated by the host immune response, and this could be one of the factors which trigger stage conversion to the bradyzoite stage [16]. In special situations, such as pregnancy, recrudescence can occur, thus bradyzoites get reactivated, and the loss of an efficient Th-1 response of a pregnant dam can lead to limited suppression of the cell mediated immunity which normally keeps tachyzoite proliferation in check [16]. This temporary break in host immunity, which normally does not cause clinical disease in the dam, can lead to transplacental infection of the fetus and abortion.

## 3. Neosporosis

In cattle, infection with *N. caninum* causes epidemic and endemic abortions. Abortions can occur from the third month of gestation to term, but evidently most of *Neospora*-induced abortions arise during mid-gestation at five to six months [17,18].

Horizontal transmission of *N. caninum* occurs by ingestion of tissue cysts, which is most likely relevant for carnivores, and by the uptake of food and water contaminated with sporulated oocysts [2]. Vertical transmission from dam to fetus can be separated into two distinct ways of infection, designated “exogenous” or “endogenous” transplacental transmission. Exogenous transplacental transmission occurs when a pregnant dam acquires the infection primarily after the ingestion of oocyst-contaminated food or water, and parasites subsequently infect the fetal tissue. Endogenous transplacental transmission is due to the recrudescence, thus reactivation and reconversion of bradyzoites into tachyzoites during pregnancy and subsequent fetal infection. Fetuses are then either aborted or born persistently infected [19]. While endogenous vertical transmission from persistently infected cattle to their offspring is believed to be mainly responsible for maintaining the parasite within a population [2,19], the presence of farm dogs might have biological relevance, because they were significantly connected to the occurrence of sero-positive cows, which in turn increased the abortion risk in a herd.

Several mechanisms might lead to abortion or fetal damage. Direct tissue damage can be caused by the multiplication of parasites in the placenta or in fetal tissues. Secondly, tissue damage can occur through the activation of the maternal immune system that elicits the production of pro-inflammatory cytokines, chemokines, nitric oxide or prostaglandins in the placenta. Subsequently, the lack of oxygen and nutrition due to placental damage might have a negative impact on fetal survival [16,17]. In general, aborted fetuses from the first trimester of pregnancy suffered from a higher parasite burden than those that had aborted in the late terms of pregnancy, where parasites could only be detected in the brain [15]. Therefore, severity and the outcome of the infection are dependent on the stage of gestation when the infection occurred. While fetal death occurs upon infection during the first trimester of pregnancy, fetal survival is more likely following infection in the last trimester.

The majority of calves (up to 95%) that are born to sero-positive cows exhibit no clinical abnormalities and are apparently healthy, but they are themselves persistently infected and will transmit the parasite to their progeny [17,20]. Very rarely, persistently infected calves develop neurological signs, and few reports documented ataxia, decreased patellar reflexes and the loss of conscious proprioception, exophthalmia, hydrocephalus or the narrowing of the spinal cord. Moreover, it was documented that some cases displayed problems to rise and were underweight compared to healthy calves [20].

Although *N. caninum* can cause repeated abortions in the same dam, the risk of abortions is reduced in subsequent gestations [15,16,21]. Therefore, some form of protective immunity exists, and a major challenge is to elucidate the basis of this protective immunity and translate these findings into effective measures that prevent infection and abortion in cattle by *N. caninum*.

## 4. Vaccines Against *Neosporosis*

There are three main options available for the cattle producer to control neosporosis: (i) test-and-cull with elimination of infected animals from the herd or from breeding; (ii) parasiticidal treatment; (iii) vaccination. The latter appeared to be the most cost-effective approach in controlling neosporosis [22,23]. Thus, there have been considerable investments in developing vaccines against *N. caninum* infection. Corresponding *in vivo* studies on host-parasite interactions and immunological responses have been mostly carried out in small laboratory model such as mice and in cattle [11]. Experimental investigations were performed in sheep (e.g., [24-28]), and also in birds (e.g., [29-31]. While studies in bovines clearly have the invaluable advantage of performing investigations in the actual target host, only few laboratories actually have the facilities to perform these studies. Thus, due to economic constraints and space-limitations, many initial experiments on vaccine candidates have been performed in small laboratory animals, mostly in mice (Table 1).

Basically, an efficient vaccine against *N. caninum* infection should fulfill the following requirements: (i) prevention of tachyzoite proliferation and dissemination in pregnant cattle (or other animals) to avoid transplacental transmission to the fetus; (ii) prevention or reduction of oocyst shedding in dogs (or other final hosts); (iii) prevention of tissue cyst formation in animals that have been infected with oocysts or tissue cysts (to avoid parasite transmission to carnivorous hosts).

This could be achieved by a vaccine that stimulates protective cellular immune responses as well as antibody responses at both, mucosal sites and systemically [11,32,33]. This is best achieved by delivering these antigens to antigen presenting cells (APCs) such as B cells and dendritic cells in a manner that allows optimal presentation via MHC class II molecules. However, experiments in cattle showed that antibody responses appear to be less important [33]. In fact, cytotoxic T cells that kill *Neospora*-infected cells in cattle are, surprisingly, CD4+-positive [34]. The T-helper cells activated by this vaccine must exert protective activities by the production of cytokines (IFN-γ, TNF-α, IL12, or IL10 and IL4, depending whether a Th1- or Th2-type immune response is elicited) and growth factors such as IL-2. Specific antibodies that are generated could aid in limiting parasite proliferation by killing extracellular tachyzoites, e.g., by interference in host cell invasion, activation of complement, and/or opsonization of parasites to have them killed by macrophages. Most importantly, since abortion can occur from chronically infected cows, immunizing an already infected cow without causing an immunological imbalance that could affect the fetus represents a major challenge.

This multitude of tasks is very unlikely to be achieved by vaccination with a single, defined antigen, but most likely with a mixture of parasite antigens, or a vaccine that contains a number of relevant antigenic domains of different proteins. These antigens are conveniently investigated in the murine model. In spite of the fact that murines and bovines exhibit somewhat different immunological characteristics, studies using mice have led to a better understanding of the complex immune response that dictates *N. caninum* infection, (reviewed in [11,15,35]). Thus, studies in mice provide a “proof-of-concept-model” and also provide important information on the immuno-protection associated with intracellular parasitism *per se*.

Protective immune responses against experimentally induced neosporosis in acute disease mouse models have been mainly associated with the development of a Th1-type immune response, dominated by IgG2a antibody production, and natural killer (NK) cell proliferation with increased IFN-γ amma production [36]. However, there are also reports on protective effects achieved by Th2-type responses in acute disease and in fetal infection models. Thus, both Th1 and Th2-driven immune mechanisms can limit disease, at least in the mouse model (reviewed in [11,35]).

### 4.1. Parasite Antigens Involved in Host Cell Invasion as Potential Vaccine Targets

Subtle but distinct variations as have been observed between *T. gondii* and *N. caninum*, can account for the differences in the biological characteristics of the two species [11,37]. However, in general, the mechanisms that govern the physical interaction between parasite and host cell are most likely largely conserved, and represent potential targets for intervention [11].

*T. gondii* and *N. caninum* actively invade a large variety of target cells. The basic mechanisms on how host cell invasion is achieved are conserved among the apicomplexans. Due to the genetic amenability and suitable animal models, *T. gondii* has served as the prime model organism to study these processes [38,39]. Surface antigens (SAGs) as well as secretory proteins, such as microneme proteins (MICs), rhoptry proteins (ROPs) and dense granule antigens (GRA proteins), represent the important key players. Host cell invasion is initiated by a primary low-affinity contact between the SAGs and host cells surface membrane. Under optimal conditions, this is then followed by a more firm apical attachment mediated by MICs. MICs originate from micronemes, small cigar-shaped organelles located at the apical part of the parasite. Many micronemal MIC-proteins exhibit conserved adhesive domains, such as thrombospondin (TSP)-like, epidermal growth factor (EGF)-like, integrin-like (I)-like, and lectin-like domains, which are known to mediate protein–protein or protein–carbohydrate interactions. MICs exist in either membrane-bound or soluble forms, and several MICs have been demonstrated to build up complexes, which are formed in the endoplasmatic reticulum, then travel through the secretory pathway, and are finally released prior to invasion [38,39]. Most likely, *Neospora* microneme proteins, in analogy to *T. gondii* and other apicomplexans are also deployed and function as protein complexes [11].

Host cell entry is achieved by the formation of a moving junction composed of MICs and ROPs, a structure between parasite and host cell surface membrane, which is reminiscent of a tight junction in mammalian cells, and which slides over the parasite as it invades its host cell [38-40]. The parasite actively moves into the host cell interior by pulling transmembrane MIC-complexes and or the moving junction towards its posterior end, and thereby invaginating the host cell surface membrane, and resealing it at its posterior end, to create a vacuole [40]. During the invasion process, rhomboid proteases are responsible for shedding of the MIC proteins from the posterior end [38-40]. The actual driving force for gliding motility is provided by a small myosin motor anchored in the inner membrane complex, whereby myosin binds to small actin filaments which are formed between inner and outer membranes [41,42]. These small actin filaments bind to the cytoplasmic domains of MIC proteins through the F-actin binding protein aldolase [43].

Once the parasites reside inside the parasitophorous vacuole, GRA proteins secreted from dense granule organelles modify the membrane of the parasitophorous vacuole, and also organize and remodel the tubular network that is built up by lipids and forms the matrix of the parasitophorous vacuole. Recent evidence suggested that the host cell is the major contributor of the lipids that build up this tubular network [44].

Taken together, these elements involved in mediating the interactions between parasites and host cell represent valuable targets for intervention through vaccination.

### 4.2. Complex and Live Attenuated Vaccines Against N. caninum Infection

#### 4.2.1. Killed Tachyzoite Lysates

The first hint that an approach employing a complex mixture of antigens could confer protection against neosporosis was provided by Liddell *et al.* [45], which showed that immunization with a killed tachyzoite lysate prevented fetal infection in Balb/c mice. Ribeiro *et al.* [46] confirmed the protective effect of a total protein lysate against cerebral infection in C57Bl/6 mice with 100% survivors after lethal challenge, whereas the excreted-secreted antigen fraction was not efficient. Vaccination with tachyzoite crude lysate was also tested in experimentally infected sheep with some degree of protection against fetal transmission. When sheep were challenged late in gestation, the number of sero-positive lambs was slightly reduced but no difference in the number of births was observed [25]. However, when sheep were challenged early in gestation the number of live-born lambs was significantly higher in the vaccinated group than in the non-vaccinated control, although the number of sero-positive lambs was the same in both groups [24].

For several years a commercial vaccine against bovine neosporosis based on killed tachyzoite lysate (Bovilis Neoguard™) has been available in a few countries. This commercial product has meanwhile been taken off the market again in the US. Clearly, protection against fetal infection with a killed tachyzoite lysate has not been achieved in cattle, but the abortion rate was reported to be reduced by approximatively 50% [47].

A formulation of total antigen extract combined with immune stimulating complex (ISCOMs) was injected into sero-negative calves and the immune response and blood parasitemia was assessed following live challenge of *N. caninum* tachyzoites. The protective effect of the vaccine was not assessed, but higher antibody titers and similar IFN-γ production were observed in those calves that had received the antigens with ISCOMs compared to those that had received live parasites as immunization dose [48]. Similarly, differences in the protective effects of a killed tachyzoite lysate were observed in the mouse model with the use of different adjuvants and in an antigen dose-dependent manner [49]. These results point out that the addition of a potent adjuvant to a killed tachyzoite extract may increase its ability to protect against neosporosis.

#### 4.2.2. Live-Attenuated Vaccines

The only vaccine against toxoplasmosis currently on the market is a live vaccine (Toxovac™), which is licensed for use in sheep in Europe and New Zealand. Toxovac™ contains live attenuated tachyzoites of the non-persistent strain *T. gondii S48* [50]. However, Toxovac™ does not protect against *N. caninum* infection and the vaccine has not been licensed in humans or other species because it is not well defined and there is the fear of reverting to tissue cyst formation. Nevertheless, others have taken over the option of vaccinating mice [51,52] or cattle [33] with live tachyzoites of the naturally attenuated low virulence *N. caninum* isolate Nc-Nowra. Challenge of these animals with the virulent Nc-Liverpool isolate resulted in infection of fetuses in only 8% of all experimentally infected mice. In addition, complete protection in cattle was achieved. This data is impressive and suggests that live vaccines, such as for toxoplasmosis in sheep, provide the best protection. Other *N. caninum* strains isolated from healthy congenitally infected calves in Spain were also shown to have low pathogenicity in experimentally infected mice [53-56] and cattle [57].

Protection against lethal challenge of virulent tachyzoites was also achieved by immunization of mice with tachyzoites attenuated by prolonged passage in tissue culture [58]. It was already previously demonstrated that protection against a lethal challenge dose could be increased after a first injection of sub-lethal dose of parasites [59] but Bartley *et al.* observed more severe pathology after the second challenge in the mice that received a sub-lethal dose of virulent tachyzoites than in the mice which were injected with the attenuated strain [58].

Other approaches included the generation of temperature-sensitive mutants of *N. caninum*, or irradiation of tachyzoites, thus treatments that lead to suppressed proliferation. Therefore, these parasites did not cause disease in mice [60,61]. Establishing a vaccine strain of *N. caninum* by genetic manipulation was also considered. Recently, Marugan-Hernandez *et al.* [62] generated a Nc-1 knock-in mutant expressing SAG4 bradyzoite protein during the tachyzoite stage. The aim was to stimulate an immune response against bradyzoite antigens at the time of infection in order to later inhibit the establishment of a chronic infection. One of the clones tested was indeed less virulent than the wild type and both parasite burden in dams brains as well as vertical transmission were reduced, suggesting that transgenic strains may be good alternatives for further safe live vaccine development.

Considering the close relationship between *N. caninum* and *Toxoplasma gondii* and taking into account the evidence of cellular and humoral cross-reactivity between the two species [63,64], the possibility of using a vaccinal strain of *T. gondii* to protect against *N. caninum* infection was investigated. While vaccination with *T. gondii* failed to prevent fetal transmission after *N. caninum* challenge in sheep [27], heterologuous vaccination using a *T. gondii* Mic1-Mic3-KO strain conferred protection against lethal challenge of *N. caninum* in mice by both oral and intraperitoneal vaccination routes with 80% and 70% survival rates, respectively. Interestingly, immunization of the mice with the Nc-1 strain conferred the same level of protection when administered orally (70% of survivors), whereas none of the mice that were injected intraperitoneally survived [65]. However, the genotypes of all these parasite populations are not well defined, and the risks associated with using live parasite populations as live vaccines in cattle may be significant, as there is the concern that these organisms might reverse to a disease-causing phenotype. In addition, reliable production of live vaccines requires a more elaborate infrastructure, vaccines have a limited shelf life, and storage is a problem. Thus, regulatory and practical hurdles to get such a vaccine marketed are high. In contrast to live vaccines, vaccines composed of recombinant proteins have distinct advantages, such as ease of manufacture, longer shelf life, ease in handling and application. It is conceivable that a focused selection of relevant antigens will also induce the relevant immune responses and reduce not only abortion, but also lead to reduced fetal infection rates.

#### 4.2.3. Subunit Vaccines

Currently, only one subunit vaccine against an apicomplexan parasite is commercially available. CoxAbic^®^ is composed of affinity-purified gametocyte antigens from *Eimeria maxima* and confers protection of hens and their offspring against coccidiosis by transmission of specific antibodies via egg yolk [66,67]. The success of these vaccines in preventing parasitic disease provide a proof that subunit vaccines can be protective and that focus on the development of recombinant subunit vaccines could be worth the trouble.

An increasing number of recombinant proteins have been investigated as vaccine candidates against neosporosis. These include mostly immune-dominant antigens functionally involved in tachyzoite-host cell interactions or surface antigens. The surface antigen NcSRS2 expressed in recombinant vaccinia virus offered adequate protection against transplacental passage and was found to limit parasite dissemination [68]. Immunization of mice against neosporosis with different recombinant NcSRS2 iscom formulations was reported to induce specific antibody responses to native NcSRS2, and a significant reduction of cerebral parasite load in immunized mice [69,70]. In fact, NcSRS2 in its native form was shown to protect against *N. caninum* congenital transmission in mice, by eliciting a Th2-type immune response [71]. However, mice vaccinated with individual recombinant recNcSRS2 and recNcSAG1 expressed in *E. coli* did not exhibit high levels of protection against challenge infection with *N. caninum* tachyzoites (30–40%), except when a combined protocol utilizing DNA vaccination and subsequent boost with the corresponding antigens was performed (75% for NcSRS2; 62.5% for NcSAG1) [72]. Reduced materno-fetal transmission was seen upon vaccination with NcGRA7 as well as NcHSP33 as a DNA vaccine [73]. Further, the immune protective effect of NcGRA7 was enhanced upon use of it as plasmid DNA together with CpG adjuvant, which improved the protective efficacy against congenital transfer [74]. Cho *et al.* [75] reported vaccination efficacy in an acute disease model upon combining recombinant NcSRS2 and dense granule antigen NcDG1. Expression of the *N. caninum* antigens NcMIC1, NcSRS2, NcGRA7 and NcGRA6, respectively, in the *Brucella abortus* vaccine strain RB51 and subsequent immunization of mice resulted in complete protection against experimentally induced acute disease and NcGRA6 and NcMIC1 expressed in *Brucella abortus* protected against vertical transmission [76,77]. Mice vaccinated with recNcMIC3 antigen exhibited high (75%) protection against cerebral infection which was associated with an IgG1 antibody response [78]. Upon recNcMIC1-vaccination and subsequent challenge infection, all mice were PCR positive in the brain, but with very low parasite burden, and recNcMIC1-vaccinated mice did not show any clinical signs of disease [79]. In contrast, DNA vaccination was not protective [79]. Intraperitoneal vaccination of mice with recNcPDI, corresponding to a protein disulfide isomerase which is partially localized in the micronemes and on the surface of *N. caninum* tachyzoites [80], induced a strong humoral immune response, but was completely ineffective in terms of inducing protective effects against *N. caninum* challenge infection. However, when recNcPDI, suspended in cholera toxin adjuvant, was administered by intranasal inoculation, there was a pronounced protective effect with 90% survivors, all with significantly decreased cerebral parasite burden [81]. A recent vaccination study comparing *Neospora* cyclophilin (NcCyp), a secretory antigen characterized as potent stimulator of host IFN-y production [82], with NcSRS2 demonstrated a high protectivity against cerebral infection, supported by high antibody titers. RecNcCyp alone conferred a level of protection that was at least as good as NcSRS2 (78.9–84.6% *vs*. 60%), whereas a combination of both antigens did not increase the protective effect [83].

ROP proteins have not been used as vaccines against *N. caninum* infection, with one exception: NcROP2. Following challenge with *N. caninum* tachyzoites, mice vaccinated with recNcROP2 remained clinically healthy and exhibited highly significantly reduced cerebral parasite burden. IgG-isotype ELISA indicated that NcROP2 induces a protective Th-1- or Th-2-biased immune response against experimental *N. caninum* infection, depending on the adjuvants (Freunds *versus* saponin) used for immunization [84]. Subsequently it was shown that a combination of recNcROP2 with recNcMIC1 and recNcMIC3 conferred a high protection against cerebral infection in experimentally infected mice without any morbidity in the mice vaccinated with the three antigens together. Moreover, the combination of the three antigens considerably reduced the vertical transmission in pregnant mice compared to the controls, whereas ROP2 alone showed the highest protection with 50% of surviving pups [85].

However, other recombinant antigens tested in vaccination studies were not efficient. Ellis *et al.* [51] reported very limited protection against transplacental transmission of *N. caninum* in mice following application of MIC10 and p24B recombinant antigens, but no protection at all using recombinant versions of NGRA1, NcGRA2, MIC10 and p24B alone. Vaccination of mice with recNcMAG1, a bradyzoite antigen, conferred no protection against cerebral infection [81]. This is not surprising, since NcMAG1 is a bradyzoite marker, which incorporates into the cyst matrix and cyst wall during tachyzoite-to bradyzoite stage conversion, and it is not likely to play an important role during acute disease [86]. Aguado-Martinez *et al.* [87] also demonstrated that vaccination of mice with NcGRA7, NcSAG4 (a bradyzoite antigen), or a combination of both, failed to protect against cerebral infection and vertical transmission, despite strong humoral and cellular immune responses. It was even demonstrated that some antigens confer anti-protective effects. Application of native, purified NcMIC4, recombinant NcMIC4 and a NcMIC4-DNA-vaccine resulted in increased cerebral parasite burden and mortality in experimentally infected mice [88].

#### 4.2.4. Amelioration of Immune Response Stimulation to Subunit Vaccines by Application of Carriers

In order to ameliorate the stimulation of the immune system during vaccination and enhance the protective effect of the administered antigens, different carriers were also studied. NcGRA7 was not effective to protect against experimental infection when used as single recombinant antigen, but the efficacy of NcGRA7 was improved by entrapping the antigen in oligomannose-coated liposomes. The vaccination resulted in an increased offspring survival rate and decreased parasite burden in the brains of dams, all associated with a strong T-helper 1 immune response [89]. Similarly, the survival rate of pups born from experimentally infected mice, which had been previously vaccinated with NcAMA-1 encapsulated in oligomannose-coated liposomes was much higher than the ones born from mice immunized with NcAMA-1 alone (60% *vs.* 8%). Again, a strong cellular immune response was observed with a significantly higher IFN-γ production by stimulated splenocytes from the mice vaccinated with liposome-entrapped NcAMA-1 than from the mice vaccinated with the protein alone [90]. Recently, a vaccination trial was performed with recNcPDI associated either to a chitosan-alginate or to a chitosan-alginate-mannose nanogel administered either intraperitoneally or intranasally. All formulations in both routes of administration gave rise to increased survival rates and reduced cerebral infections. However, no significant difference was observed between the groups vaccinated with the nanogels combined with the protein and the nanogels alone, suggesting a strong stimulation of the innate immune response by the nanogel itself [91]. Nevertheless, if a strong antigen-specific adaptive immune response is associated with the stimulating effect of the nanogel, this type of carrier could be of great interest for further vaccination studies.

Although none of the subunit vaccines developed so far was able to prevent the vertical transmission of *N. caninum* with a sufficiently high efficacy to be applied on the market, some showed promising results and further research should be carried out. New methods that permit to identify some putative protective antigens such as genetic mapping may help perform a meaningful selection. A recent study pointed out that only few antigens were relevant for the protection against *Eimeria sp.*, with one of them, IMP-1, that has a homologue in the *N. caninum* and *T. gondii* genome [92].

#### 4.2.5. Chimeric Vaccines

In malaria, caused by *Plasmodium* parasites, vaccination with chimeric vaccines bearing several immunogenic epitopes has been used since the 1980s. A number of such antigens are currently in clinical trials, such as a vaccine against *P. falciparum* composed of AMA1/MSP1 chimeric protein [93-95]. Although a number of poly-epitope chimeric antigens incorporating multiple protective epitopes from multiple antigens and life cycle stages have been considered to be more effective than single stage vaccines, the desired outcome is not always achieved, and the success and efficiency is highly dependent on the configuration of the chimeric antigenic molecule [96,97].

Chimeric antigens have also been used experimentally to prevent infection with other apicomplexans such as *Toxoplasma* and *Eimeria*. By combining recombinant TgROP2 with *Leishmania infantum* Hsp83 as a fusion protein, Echeverria *et al.* [98] showed that protection was significantly increased compared to immunization with recombinant TgROP2 alone. Another combined vaccine was generated by Zhang *et al.* [99], who combined TgROP2 and TgSAG1 as a chimeric DNA vaccine, and inoculated it into Balb/c mice with and without a plasmid encoding murine IL-12. The chimeric TgROP2-TgSAG1 plasmid combined with pIL-12 significantly delayed mortality in mice challenged with RH-strain *T. gondii*. The immune response generated by the TgROP2-SAG1 DNA vaccine was also compared to an immunization of mice with a TgROP2-SAG1 recombinant protein or a vaccination combining both strategies. A strong humoral immune response was observed after vaccination with the recombinant fusion protein whereas a rather cellular biased immune response was observed after DNA vaccination, suggesting that a mixed immunization using both DNA and recombinant proteins may be of interest to stimulate both Th-1 and Th-2 T-cells subtypes [100]. Another report by Gatkowska *et al.* [101] demonstrated the use of chimeric *E. coli* Dr fimbriae, bearing selected antigenic epitopes of TgSAG1 (expressed in tachyzoites), MAG1 (expressed in tachyzoites and bradyzoites), and GRA1 (expressed in both stages). The recombinant chimeric antigen induced a strong antibody response and a brain cyst reduction by 89%. Vaccination of chickens with a chimeric DNA vaccine encoding *E. tenella* TA4 and chicken IL-2 induced protective immunity in chickens [102].

Thus, chimeric vaccines should also be considered as tools for preventing neosporosis in cattle. This approach was applied by Monney *et al.* [103]. Since a mixture of three recombinant antigens representing NcMIC1, NcMIC3 and NcROP2 was shown to confer a high degree of protection in both cerebral and fetal infection models in mice [85], Monney *et al.* selected immunodominant domains of each of these proteins employing a number of bioinformatic tools, and created four different chimeric antigens, with the respective domains incorporated into these chimers in different orders. Following vaccination, challenged mice were carefully monitored for clinical symptoms during four weeks post-infection. Of the four chimeric antigens, only one, named MIC3-1-R, provided complete protection against disease with 100% survivors, compared to 40–80% of survivors in the other groups. Only sera of MIC3-1-R vaccinated mice, but not the others, recognized all three proteins on Western blots of *N. caninum* extracts. However, whether this is the reason why protection was achieved with only MIC3-1R and not with other chimeric antigens has not yet been elucidated. Serology did not show any clear differences in total IgG, IgG1 and IgG2a levels between the different treatment groups. Vaccination with all four chimeric variants generated a rather IL-4 biased cytokine expression, which then shifted to an IFN-γ-dominated response following experimental infection. These results indicate that chimeric antigens can confer protection against cerebral disease, and should be followed up in subsequent studies in a fetal infection model.

## 5. Conclusions

An efficacious and safe vaccine that limits congenital infection of cattle with *N. caninum* is not on the market. Based on the very good protection rates achieved by live vaccination with the naturally attenuated NC-Nowra isolate, and the still relatively modest success employing recombinant vaccines, live vaccines could be one avenue to go along. However, live vaccines have some serious disadvantages in terms of safety, costs of production, and stability of the final product.

In contrast, subunit vaccines have clear advantages such as reduced costs in production, processing and storage, increased stability and shelf life. The current evidence obtained from studies in the mouse model suggests that a suitable subunit vaccine could target proteins involved in the interaction of parasites with the host cell. Clearly, more basic and independent research on the molecular nature and function of selected *Neospora* antigens is needed in order to evaluate their true usefulness as potential vaccine candidates.

A highly valuable advantage in *Neospora* research has been the fact that the current knowledge on the cell biology, and especially the molecular aspects of host-parasite interactions, obtained from other apicomplexan models such as *Toxoplasma*, were extrapolated to the situation in *Neospora*. However, in many cases this has been done without carefully evaluating potential differences between the two species. Therefore, it is important to elucidate what *Neospora* is doing differently compared to *Toxoplasma*, and how we could exploit these differences to generate an efficient vaccine (or any other tool of intervention).

As different groups employ different animal models, routes of infection and different parameters for assessment of vaccines, it is difficult to directly compare the results coming from different laboratories, and a certain level of standardization would represent a great improvement. In addition, there is a great need for the development of a laboratory recrudescence model, which would mimic the situation of endogenous transplacental infection as it occurs in cattle in the field. However, there are admittedly substantial differences in the inherent properties of the immune responses between mice and cattle, and any results derived in a small animal laboratory model, however promising they are, need to be evaluated with caution.

## Figures and Tables

**Table 1:** Summary of vaccine candidates (killed lysates, live-attenuated, recombinant subunit vaccines) and animal models investigated for the prevention of *N. caninum* infection. rec = recombinant; live = live vaccine; MIC = microneme antigen; PDI = protein disulfide isomerase; SAG = surface antigen; GRA = dense granule antigen; MAG = matrix-asociated antigen; ROP = rhoptry antigen; SRS = SAG1-related surface antigen; DG1 = dense granule antigen 1; HSP = heat shock protein.

**Vaccine candidates**	**Protection**	**Animal model**	**References**
Chimeric vaccine MIC1-3-R rec	+	mice	[103]
PDI rec	+	mice	[81,89]
SAG4 + GRA7 rec	−	mice	[87]
MAG1 rec	−	mice	[79]
GRA1, GRA2, MIC10, p24B rec	−	mice	[51]
MIC4 rec	−	mice	[88]
Cyclophillin + SRS2 rec	+	mice	[83]
MIC1, MIC3, ROP2 rec	+/−	mice	[78,79,84]
ROP2 + MIC1 + MIC3 rec combined	+	mice	[85]
NcGRA6, NcMIC1 in *B. abortus*	+	mice	[76,77]
SRS2 + DG1 rec combination	+	gerbil	[75]
GRA7, HSP33 rec	+/−	mice	[73,74]
SAG1, SRS2 rec	+/−	mice	[72]
SRS2 rec or native	+	mice	[68-71]
MIC1 + MIC3 KO *T. gondii* live	+	mice	[65]
Live attenuated *N. caninum*	+	mice	[51,52,58,60-62]
Live attenuated *N. caninum*	+	cattle	[33]
Killed *N. caninum* tachyzoite lysate	+/−	cattle	[33,47]
*T. gondii* live	−	sheep	[27]
Killed *N. caninum* tachyzoite lysate	+/−	sheep	[24,25]
Killed *N. caninum* tachyzoite lysate	+	mice	[45,46]

## References

[1] Dubey J.P., Carpenter J.L., Speer C.A., Topper M.J., Uggla A. (1988). Newly recognized fatal protozoan disease of dogs. J. Am. Vet. Med. Assoc..

[2] Dubey J.P., Schares G., Ortega-Mora L.M. (2007). Epidemiology and control of neosporosis and *Neospora caninum*. Clin. Microbiol. Rev..

[3] Bartels C.J., Arnaiz-Seco J.I., Ruiz-Santa-Quitera A., Björkman C., Frössling J., von Blumröder D., Conraths F.J., Schares G., van Maanen C., Wouda W. (2006). Supranational comparison of *Neospora caninum* seroprevalences in cattle in Germany, The Netherlands, Spain and Sweden. Vet. Parasitol..

[4] Reichel M.P. (2000). *Neospora caninum* infections in Australia and New Zealand. Aust. Vet. J..

[5] Wang C., Wang Y., Zou X., Zhai Y., Gao J., Hou M., Zhu X.Q. (2010). Seroprevalence of *Neospora caninum* infection in dairy cattle in northeastern China. J. Parasitol..

[6] Gottstein B., Hentrich B., Wyss R., Thür B., Busato A., Stärk K.D., Müller N. (1998). Molecular and immunodiagnostic investigations on bovine neosporosis in Switzerland. Int. J. Parasitol..

[7] Dubey J.P., Schares G. (2011). Neosporosis in animals-The last five years. Vet. Parasitol..

[8] Pfeiffer D.U., Williamson N.B., Reichel M.P., Wichtel J.J., Teague W.R. (2002). A longitudinal study of *Neospora caninum* infection on a dairy farm in New Zealand. Prev. Vet. Med..

[9] Häsler B., Stärk K.D., Sager H., Gottstein B., Reist M. (2006). Simulating the impact of four control strategies on the population dynamics of *Neospora caninum* infection in Swiss dairy cattle. Prev. Vet. Med..

[10] Häsler B., Regula G., Stärk K.D., Sager H., Gottstein B., Reist M. (2006). Financial analysis of various strategies for the control of *Neospora caninum* in dairy cattle in Switzerland. Prev. Vet. Med..

[11] Hemphill A., Vonlaufen N., Naguleswaran A. (2006). Cellular and immunological basis of the host-parasite relationship during infection with *Neospora caninum*. Parasitology.

[12] Dubey J.P., Jenkins M.C., Rajendran C., Miska K., Ferreira L.R., Martins J., Kwok O.C., Choudhary S. (2011). Gray wolf (*Canis lupus*) is a natural definitive host for *Neospora caninum*. Vet Parasitol..

[13] Strohbusch M., Müller N., Hemphill A., Margos M., Grandgirard D., Leib S., Greif G., Gottstein B. (2009). *Neospora caninum* and bone marrow-derived dendritic cells: parasite survival, proliferation, and induction of cytokine expression. Parasite Immunol..

[14] Dion S., Germon S., Guiton R., Ducournau C., Dimier-Poisson I. (2011). Functional activation of T cells by dendritic cells and macrophages exposed to the intracellular parasite *Neospora caninum*. Int. J. Parasitol..

[15] Innes E.A. (2007). The host-parasite relationship in pregnant cattle infected with *Neospora caninum*. Parasitology.

[16] Innes E.A., Bartley P.M., Maley S.W., Wright S.E., Buxton D. (2007). Comparative host-parasite relationships in ovine toxoplasmosis and bovine neosporosis and strategies for vaccination. Vaccine.

[17] Buxton D., McAllister M.M., Dubey J.P. (2002). The comparative pathogenesis of neosporosis. Trends Parasitol..

[18] Dubey J.P. (2003). Review of *Neospora caninum* and neosporosis in animals. Korean J. Parasitol..

[19] Williams D.J., Hartley C.S., Björkman C., Trees A.J. (2009). Endogenous and exogenous transplacental transmission of *Neospora caninum*—How the route of transmission impacts on epidemiology and control of disease. Parasitology.

[20] Dubey J.P., Buxton D., Wouda W. (2006). Pathogenesis of bovine neosporosis. J. Comp. Pathol..

[21] Thurmond M.C., Hietala S.K. (1997). Effect of congenitally acquired *Neospora caninum* infection on risk of abortion and subsequent abortions in dairy cattle. Am. J. Vet. Res..

[22] Reichel M.P., Ellis J.T. (2006). If control of *Neospora caninum* infection is technically feasible does it make economic sense?. Vet. Parasitol.

[23] Reichel M.P., Ellis J.T. (2008). Re-evaluating the economics of neosporosis control. Vet. Parasitol..

[24] Jenkins M.C., Tuo W., Dubey J.P. (2004). Evaluation of vaccination with *Neospora caninum* protein for prevention of fetal loss associated with experimentally induced neosporosis in sheep. Am. J. Vet. Res..

[25] O'Handley R.M., Morgan S.A., Parker C., Jenkins M.C., Dubey J.P. (2003). Vaccination of ewes for prevention of vertical transmission of *Neospora caninum*. Am. J. Vet. Res..

[26] O'Handley R., Liddell S., Parker C., Jenkins M.C., Dubey J.P. (2002). Experimental infection of sheep with *Neospora caninum* oocysts. J. Parasitol..

[27] Innes E.A., Lundén A., Esteban I., Marks J., Maley S., Wright S., Rae A., Harkins D., Vermeulen A., McKendrick I.J., Buxton D. (2001). A previous infection with *Toxoplasma gondii* does not protect against a challenge with *Neospora caninum* in pregnant sheep. Parasite Immunol..

[28] Buxton D., Wright S., Maley S.W., Rae A.G., Lundén A., Innes E.A. (2001). Immunity to experimental neosporosis in pregnant sheep. Parasite Immunol..

[29] Mansourian M., Khodakaram-Tafti A., Namavari M. (2009). Histopathological and clinical investigations in *Neospora caninum* experimentally infected broiler chicken embryonated eggs. Vet. Parasitol..

[30] Mineo T.W., Carrasco A.O., Marciano J.A., Werther K., Pinto A.A., Machado R.Z. (2009). Pigeons (*Columba livia*) are a suitable experimental model for *Neospora caninum* infection in birds. Vet. Parasitol..

[31] Furuta P.I., Mineo T.W., Carrasco A.O., Godoy G.S., Pinto A.A., Machado R.Z. (2007). *Neospora caninum* infection in birds: experimental infections in chicken and embryonated eggs. Parasitology.

[32] Eperon S., Brönnimann K., Hemphill A., Gottstein B. (1999). Susceptibility of B-cell deficient C57BL/6 (microMT) mice to *Neospora caninum* infection. Parasite Immunol..

[33] Williams D.J., Guy C.S., Smith R.F., Ellis J., Björkman C., Reichel M.P., Trees A.J. (2007). Immunization of cattle with live tachyzoites of *Neospora caninum* confers protection against fetal death. Infect. Immun..

[34] Staska L.M., McGuire T.C., Davies C.J., Lewin H.A., Baszler T.V. (2003). *Neospora caninum*-infected cattle develop parasite-specific CD4+ cytotoxic T lymphocytes. Infect. Immun..

[35] Reichel M.P., Ellis J.T. (2009). *Neospora caninum*—How close are we to development of an efficacious vaccine that prevents abortion in cattle?. Int. J. Parasitol..

[36] Khan I.A., Schwartzman J.D., Fonseka S., Kasper L.H. (1997). *Neospora caninum*: Role for immune cytokines in host immunity. Exp. Parasitol..

[37] Innes E.A., Mattsson J.G. (2007). *Neospora caninum* emerges from the shadow of Toxoplasma gondii. Trends Parasitol..

[38] Carruthers V., Boothroyd J.C. (2007). Pulling together: An integrated model of *Toxoplasma* cell invasion. Curr. Opin. Microbiol..

[39] Sibley L.D. (2011). Invasion and intracellular survival by protozoan parasites. Immunol. Rev..

[40] Sibley L.D. (2004). Intracellular parasite invasion strategies. Science.

[41] Meissner M., Schlüter D., Soldati D. (2002). Role of *Toxoplasma gondii* myosin A in powering parasite gliding and host cell invasion. Science.

[42] Sahoo N., Beatty W., Heuser J., Sept D., Sibley L.D. (2006). Unusual kinetic and structural properties control rapid assembly and turnover of actin in the parasit *Toxoplasma gondii*. Mol. Biol. Cell..

[43] Starnes G.L., Coincon M., Sygusch J., Sibley L.D. (2009). Aldolase is essential for energy production and bridging adhesin-actin cytoskeletal interactions during parasite invasion of host cells. Cell Host Microbe.

[44] Caffaro C.E., Boothroyd J.C. (2011). Evidence for host as the major contributor to lipid in the intravacuolar network of toxoplasma-infected cells. Eukaryot. Cell.

[45] Liddell S., Jenkins M.C., Collica C.M., Dubey J.P. (1999). Prevention of vertical transfer of *Neospora caninum* in BALB/c mice by vaccination. J. Parasitol..

[46] Ribeiro D.P., Freitas M.M., Cardoso M.R., Pajuaba A.C., Silva N.M., Mineo T.W., Silva J.S., Mineo J.R., Silva D.A. (2009). CpG-ODN combined with *Neospora caninum* lysate, but not with excreted-secreted antigen, enhances protection against infection in mice. Vaccine.

[47] Romero J.J., Pérez E., Frankena K. (2004). Effect of a killed whole *Neospora caninum* tachyzoite vaccine on the crude abortion rate of Costa Rican dairy cows under field conditions. Vet. Parasitol..

[48] Moore D.P., Echaide I., Verna A.E., Leunda M.R., Cano A., Pereyra S., Zamorano P.I., Odeón A.C., Campero C.M. (2011). Immune response to *Neospora caninum* native antigens formulated with immune stimulating complexes in calves. Vet. Parasitol..

[49] Rojo-Montejo S., Collantes-Fernandez E., Regidor-Cerrillo J., Rodriguez-Bertos A., Prenafeta A., Gomez-Bautista M., Ortega-Mora L.M. (2011). Influence of adjuvant and antigen dose on protection induced by an inactivated whole vaccine against *Neospora caninum* infection in mice. Vet. Parasitol..

[50] Buxton D. (1993). Toxoplasmosis: The first commercial vaccine. Parasitol. Today.

[51] Ellis J., Miller C., Quinn H., Ryce C., Reichel M.P. (2008). Evaluation of recombinant proteins of *Neospora caninum* as vaccine candidates (in a mouse model). Vaccine.

[52] Miller C., Quinn H., Ryce C., Reichel M.P., Ellis J.T. (2005). Reduction in transplacental transmission of *Neospora caninum* in outbred mice by vaccination. Int. J. Parasitol..

[53] Regidor-Cerrillo J., Gomez-Bautista M., Pereira-Bueno J., Aduriz G., Navarro-Lozano V., Risco-Castillo V., Fernandez-Garcia A., Pedraza-Diaz S., Ortega-Mora L.M. (2008). Isolation and genetic characterization of *Neospora caninum* from asymptomatic calves in Spain. Parasitology.

[54] Pereira Garcia-Melo D., Regidor-Cerrillo J., Collantes-Fernandez E., Aguado-Martinez A., Del Pozo I., Minguijon E., Gomez-Bautista M., Aduriz G., Ortega-Mora L.M. (2009). Pathogenic characterization in mice of *Neospora caninum* isolates obtained from asymptomatic calves. Parasitology.

[55] Rojo-Montejo S., Collantes-Fernandez E., Regidor-Cerrillo J., Alvarez-Garcia G., Marugan-Hernandez V., Pedraza-Diaz S., Blanco-Murcia J., Prenafeta A., Ortega-Mora L.M. (2009). Isolation and characterization of a bovine isolate of *Neospora caninum* with low virulence. Vet. Parasitol..

[56] Regidor-Cerrillo J., Gomez-Bautista M., Del Pozo I., Jimenez-Ruiz E., Aduriz G., Ortega-Mora L.M. (2010). Influence of *Neospora caninum* intra-specific variability in the outcome of infection in a pregnant BALB/c mouse model. Vet. Res..

[57] Rojo-Montejo S., Collantes-Fernandez E., Blanco-Murcia J., Rodriguez-Bertos A., Risco-Castillo V., Ortega-Mora L.M. (2009). Experimental infection with a low virulence isolate of *Neospora caninum* at 70 days gestation in cattle did not result in foetopathy. Vet. Res..

[58] Bartley P.M., Wright S., Chianini F., Buxton D., Innes E.A. (2008). Inoculation of Balb/c mice with live attenuated tachyzoites protects against a lethal challenge of *Neospora caninum*. Parasitology.

[59] Lunden A., Wright S., Allen J.E., Buxton D. (2002). Immunisation of mice against neosporosis. Int. J. Parasitol..

[60] Dreier K.J., Stewarter L.W., Kerlin R.L., Ritter D.M., Brake D.A. (1999). Phenotypic characterisation of a *Neospora caninum* temperature-sensitive strain in normal and immunodeficient mice. Int. J. Parasitol..

[61] Ramamoorthy S., Lindsay D.S., Schurig G.G., Boyle S.M., Duncan R.B., Vemulapalli R., Sriranganathan N. (2006). Vaccination with gamma-irradiated *Neospora caninum* tachyzoites protects mice against acute challenge with N. caninum. J. Eukaryot. Microbiol..

[62] Marugán-Hernández V., Ortega-Mora L.M., Aguado-Martínez A., Alvarez-García G. (2011). Genetic manipulation of *Neospora caninum* to express the bradyzoite-specific protein NcSAG4 in tachyzoites. Parasitology.

[63] Kasper L.H., Khan I.A. (1998). Antigen-specific CD8+ T cells protect against lethal toxoplasmosis in mice infected with Neospora caninum. Infect. Immun..

[64] Nishikawa Y., Claveria F.G., Fujisaki K., Nagasawa H. (2002). Studies on serological cross-reaction of *Neospora caninum* with *Toxoplasma gondii* and *Hammondia heydorni*. J. Vet. Med. Sci..

[65] Penarete-Vargas D.M., Mevelec M.N., Dion S., Seche E., Dimier-Poisson I., Fandeur T. (2010). Protection against lethal *Neospora caninum* infection in mice induced by heterologous vaccination with a mic1 mic3 knockout *Toxoplasma gondii* strain. Infect. Immun..

[66] Sharman P.A., Smith N.C., Wallach M.G., Katrib M. (2010). Chasing the golden egg: Vaccination against poultry coccidiosis. Parasite Immunol..

[67] Wallach M.G., Ashash U., Michael A., Smith N.C. (2008). Field application of a subunit vaccine against an enteric protozoan disease. PLoS One.

[68] Nishikawa Y., Xuan X., Nagasawa H., Igarashi I., Fujisaki K., Otsuka H., Mikami T. (2001). Prevention of vertical transmission of *Neospora caninum* in BALB/c mice by recombinant vaccinia virus carrying NcSRS2 gene. Vaccine.

[69] Pinitkiatisakul S., Friedman M., Wikman M., Mattsson J.G., Lövgren-Bengtsson K., Ståhl S., Lundén A. (2007). Immunogenicity and protective effect against murine cerebral neosporosis of recombinant NcSRS2 in different iscom formulations. Vaccine.

[70] Pinitkiatisakul S., Mattsson J.G., Wikman M., Friedman M., Bengtsson K.L., Ståhl S., Lundén A. (2005). Immunisation of mice against neosporosis with recombinant NcSRS2 iscoms. Vet. Parasitol..

[71] Haldorson G.J., Mathison B.A., Wenberg K., Conrad P.A., Dubey J.P., Trees A.J., Yamane I., Baszler T.V. (2005). Immunization with native surface protein NcSRS2 induces a Th2 immune response and reduces congenital *Neospora caninum* transmission in mice. Int. J. Parasitol..

[72] Cannas A., Naguleswaran A., Muller N., Eperon S., Gottstein B., Hemphill A. (2003). Vaccination of mice against experimental *Neospora caninum* infection using NcSAG1- and NcSRS2-based recombinant antigens and DNA vaccines. Parasitology.

[73] Liddell S., Parker C., Vinyard B., Jenkins M., Dubey J.P. (2003). Immunization of mice withplasmid DNA coding for NcGRA7 or NcsHSP33 confers partial protection against vertical transmission of Neospora caninum. J. Parasitol..

[74] Jenkins M., Parker C., Tuo W., Vinyard B., Dubey J.P. (2004). Inclusion of CpG adjuvant with plasmid DNA coding for NcGRA7 improves protection against congenital neosporosis. Infect. Immun..

[75] Cho J.H., Chung W.S., Song K.J., Na B.K., Kang S.W., Song C.Y., Kim T.S. (2005). Protective efficacy of vaccination with *Neospora caninum* multiple recombinant antigens against experimental *Neospora caninum* infection. Korean J. Parasitol..

[76] Ramamoorthy S., Sanakkayala N., Vemulapalli R., Jain N., Lindsay D.S., Schurig G.S., Boyle S.M., Sriranganathan N. (2007). Prevention of vertical transmission of *Neospora caninum* in C57BL/6 mice vaccinated with *Brucella abortus* strain RB51 expressing *N. caninum* protective antigens. Int. J. Parasitol..

[77] Ramamoorthy S., Sanakkayala N., Vemulapalli R., Duncan R.B., Lindsay D.S., Schurig G.S., Boyle S.M., Kasimanickam R., Sriranganathan N. (2007). Prevention of lethal experimental infection of C57BL/6 mice by vaccination with *Brucella abortus* strain RB51 expressing *Neospora caninum* antigens. Int. J. Parasitol..

[78] Cannas A., Naguleswaran A., Muller N., Gottstein B., Hemphill A. (2003). Reduced cerebral infection of *Neospora caninum*-infected mice after vaccination with recombinant microneme protein NcMIC3 and ribi adjuvant. J. Parasitol..

[79] Alaeddine F., Keller N., Leepin A., Hemphill A. (2005). Reduced infection and protection from clinical signs of cerebral neosporosis in C57BL/6 mice vaccinated with recombinant microneme antigen NcMIC1. J. Parasitol..

[80] Naguleswaran A., Alaeddine F., Guionaud C., Vonlaufen N., Sonda S., Jenoe P., Mevissen M., Hemphill A. (2005). *Neospora caninum* protein disulfide isomerase is involved in tachyzoite-host cell interaction. Int. J. Parasitol..

[81] Debache K., Guionaud C., Alaeddine F., Hemphill A. (2010). Intraperitoneal and intra-nasal vaccination of mice with three distinct recombinant *Neospora caninum* antigens results in differential effects with regard to protection against experimental challenge with *Neospora caninum* tachyzoites. Parasitology.

[82] Tuo W., Fetterer R., Jenkins M., Dubey J.P. (2005). Identification and characterization of *Neospora caninum* cyclophilin that elicits gamma interferon production. Infect. Immun..

[83] Tuo W., Zhao Y., Zhu D., Jenkins M.C. (2011). Immunization of female BALB/c mice with *Neospora* cyclophilin and/or NcSRS2 elicits specific antibody response and prevents against challenge infection by *Neospora caninum*. Vaccine.

[84] Debache K., Guionaud C., Alaeddine F., Mevissen M., Hemphill A. (2008). Vaccination of mice with recombinant NcROP2 antigen reduces mortality and cerebral infection in mice infected with *Neospora caninum* tachyzoites. Int. J. Parasitol..

[85] Debache K., Alaeddine F., Guionaud C., Monney T., Muller J., Strohbusch M., Leib S.L., Grandgirard D., Hemphill A. (2009). Vaccination with recombinant NcROP2 combined with recombinant NcMIC1 and NcMIC3 reduces cerebral infection and vertical transmission in mice experimentally infected with *Neospora caninum* tachyzoites. Int. J. Parasitol..

[86] Guionaud C., Hemphill A., Mevissen M., Alaeddine F. (2010). Molecular characterization of *Neospora caninum* MAG1, a dense granule protein secreted into the parasitophorous vacuole, and associated with the cyst wall and the cyst matrix. Parasitology.

[87] Aguado-Martinez A., Alvarez-Garcia G., Fernandez-Garcia A., Risco-Castillo V., Marugan-Hernandez V., Ortega-Mora L.M. (2009). Failure of a vaccine using immunogenic recombinant proteins rNcSAG4 and rNcGRA7 against neosporosis in mice. Vaccine.

[88] Srinivasan S., Mueller J., Suana A., Hemphill A. (2007). Vaccination with microneme protein NcMIC4 increases mortality in mice inoculated with *Neospora caninum*. J. Parasitol..

[89] Nishikawa Y., Zhang H., Ikehara Y., Kojima N., Xuan X., Yokoyama N. (2009). Immunization with oligomannose-coated liposome-entrapped dense granule protein 7 protects dams and offspring from *Neospora caninum* infection in mice. Clin. Vaccine Immunol..

[90] Zhang H., Nishikawa Y., Yamagishi J., Zhou J., Ikehara Y., Kojima N., Yokoyama N., Xuan X. (2010). *Neospora caninum*: Application of apical membrane antigen 1 encapsulated in the oligomannose-coated liposomes for reduction of offspring mortality from infection in BALB/c mice. Exp. Parasitol..

[91] Debache K., Kropf C., Schutz C.A., Harwood L.J., Kauper P., Monney T., Rossi N., Laue C., McCullough K.C., Hemphill A. (2011). Vaccination of mice with chitosan nanogel-associated recombinant NcPDI against challenge infection with *Neospora caninum* tachyzoites. Parasite Immunol..

[92] Blake D.P., Billington K.J., Copestake S.L., Oakes R.D., Quail M.A., Wan K.L., Shirley M.W., Smith A.L. (2011). Genetic mapping identifies novel highly protective antigens for an apicomplexan parasite. PLoS Pathog..

[93] Malkin E., Hu J., Li Z., Chen Z., Bi X., Reed Z., Dubovsky F., Liu J., Wang Q., Pan X. (2008). A phase 1 trial of PfCP2.9: An AMA1/MSP1 chimeric recombinant protein vaccine for *Plasmodium falciparum* malaria. Vaccine.

[94] Peng H., Hu Y., Zhou A., Jin C., Pan W. (2010). Solution structure of a *Plasmodium falciparum* AMA-1/MSP 1 chimeric protein vaccine candidate (PfCP-2.9) for malaria. Malar. J..

[95] Xue X., Ding F., Zhang Q., Pan X., Qu L., Pan W. (2010). Stability and potency of the *Plasmodium falciparum* MSP1-19/AMA-1(III) chimeric vaccine candidate with Montanide ISA720 adjuvant. Vaccine.

[96] Cai Q., Peng G., Bu L., Lin Y., Zhang L., Lustigmen S., Wang H. (2007). Immunogenicity and *in vitro* protective efficacy of a polyepitope *Plasmodium falciparum* candidate vaccine constructed by epitope shuffling. Vaccine.

[97] Shi Q., Lynch M.M., Romero M., Burns J.M. (2007). Enhanced protection against malaria by a chimeric merozoite surface protein vaccine. Infect. Immun..

[98] Echeverria P.C., de Miguel N., Costas M., Angel S.O. (2006). Potent antigen-specific immunity to *Toxoplasma gondii* in adjuvant-free vaccination system using Rop2-*Leishmania infantum* Hsp83 fusion protein. Vaccine.

[99] Zhang J., He S., Jiang H., Yang T., Cong H., Zhou H., Zhang J., Gu Q., Li Y., Zhao Q. (2007). Evaluation of the immune response induced by multiantigenic DNA vaccine encoding SAG1 and ROP2 of *Toxoplasma gondii* and the adjuvant properties of murine interleukin-12 plasmid in BALB/c mice. Parasitol. Res..

[100] Li W.S., Chen Q.X., Ye J.X., Xie Z.X., Chen J., Zhang L.F. (2011). Comparative evaluation of immunization with recombinant protein and plasmid DNA vaccines of fusion antigen ROP2 and SAG1 from *Toxoplasma gondii* in mice: cellular and humoral immune responses. Parasitol. Res..

[101] Gatkowska J., Gasior A., Kur J., Dlugonska H. (2008). *Toxoplasma gondii*: Chimeric Dr fimbriae as a recombinant vaccine against toxoplasmosis. Exp. Parasitol..

[102] Xu Q., Song X., Xu L., Yan R., Shah M.A., Li X. (2008). Vaccination of chickens with a chimeric DNA vaccine encoding *Eimeria tenella* TA4 and chicken IL-2 induces protective immunity against coccidiosis. Vet. Parasitol..

[103] Monney T., Rütti D., Schorer M., Debache K., Grandgirard D., Leib S.L., Hemphill A. (2011). RecNcMIC3-1-R is a microneme- and rhoptry-based chimeric antigen that protects against acute neosporosis and limits cerebral parasite load in the mouse model for *Neospora caninum* infection. Vaccine.

